# Lipidomic Insight Across 48 *Saccharomyces cerevisiae* Wine Yeast Strains Revealed Novel N-Acyl Lysophosphatidylethanolamines

**DOI:** 10.3390/microorganisms14061260

**Published:** 2026-06-03

**Authors:** Md Abdul Malek, Jayashankar Jayaprakash, Kevin S. Huynh, Divyavani Gowda, Teruo Sone, Shu-Ping Hui, Siddabasave Gowda B. Gowda

**Affiliations:** 1 Graduate School of Global Food Resources, Hokkaido University, Kita-9, Nishi-9, Kita-Ku, Sapporo 060-0809, Japan; mdabdul.malek.v0@elms.hokudai.ac.jp (M.A.M.); jayashankar.virat@gmail.com (J.J.); helloimkevin@gmail.com (K.S.H.); sonet@agr.hokudai.ac.jp (T.S.); 2 Faculty of Health Sciences, Hokkaido University, Kita-12, Nishi-5, Kita-Ku, Sapporo 060-0812, Japan; divyavani@hs.hokudai.ac.jp; 3 Faculty of Agriculture, Hokkaido University, Kita-9, Nishi-9, Kita-Ku, Sapporo 060-0809, Japan

**Keywords:** *Saccharomyces cerevisiae*, yeast, liquid chromatography, mass spectrometry, LNAPEs

## Abstract

*Saccharomyces cerevisiae* (SC), an important unicellular yeast species in the biotechnology, beverage, and food industries, has remarkable applications in ethanol production. Lipids in SC play a crucial role in stress management during the fermentation process. Despite the importance of SC yeasts, comprehensive lipid profiling across multiple yeast strains remains limited. This study aimed to elucidate the comprehensive lipidomic profiles of 48 wine yeast strains of the SC species using a nontargeted liquid chromatography–mass spectrometry approach. A total of 135 lipid species were identified, representing five major lipid categories. Among the SC strains, strain 19 contained the highest relative concentrations of α-linolenic acid (ω-3) and linoleic acid (ω-6). Levels of polyunsaturated fatty acids were 4.6-fold higher in SC46 compared to SC13, suggesting strain-specific variations. Glycerolipids were the most abundant lipid components, with triacylglycerols showing the highest relative abundance in SC10, SC41, and SC48. These findings should be interpreted in light of the semi-quantitative nature of the dataset. Interestingly, bioactive lipids, such as N-acyl lysophosphatidylethanolamines, were putatively identified and structurally characterized using high-resolution mass spectra and MS/MS fragmentation patterns. This study provides insight into strain-specific differences in the yeast lipidome and may help improve our understanding of fermentation kinetics and industrial wine quality.

## 1. Introduction

Lipids are fundamental biomolecules in eukaryotic cells that govern the membrane structure, cellular activity, nutrient transport, and stress tolerance [1,2]. *Saccharomyces cerevisiae* (SC), commonly known as budding yeast, has the remarkable ability to withstand high levels of ethanol, which potentially influences its biological and physiological properties [3]. Various yeast strains can alter ethanol tolerance, membrane fluidity, and secondary metabolite production during fermentation by adapting to diverse environmental conditions [2,4,5]. The budding yeast SC is an important model organism in many fields, including genetics, cell biology, and metabolism [6]. SC is one of the primary unicellular yeast species in the biotechnological, beverage, and food industries, and has significant applications in ethanol production [5]. Wine yeast strains are particularly important because of their ability to efficiently ferment high-fructose grape musts and tolerate rising ethanol concentrations, which directly determines fermentation efficiency and wine quality, with specific membrane lipid compositions, such as higher phosphatidylcholine, ergosterol, and unsaturated fatty acids, correlating strongly with ethanol tolerance and maximal cell growth. These traits make wine yeasts both a practical target for strain selection or engineering and a valuable model for studying how membrane lipid remodeling mitigates ethanol- and osmotic-stress during industrial fermentations [7,8]. Metabolic engineering of SC may lead to the novel production of high-value lipid-based compounds, such as sterols, steroid hormones, and polyunsaturated fatty acids, including non-native ω-3 and ω-6 [9]. Most yeast strains contain neutral lipids, primarily triacylglycerols (TGs), sterol esters, and free fatty acids. Typically, TGs are the predominant lipids, whereas other lipids may prevail in smaller amounts [10]. Yeast shows significant variations in its metabolism depending on the growing environment and changes in lipid function and structure [11]. SC has been shown to adapt dynamically to different growth conditions, such as growth on oleate, which alters lipid storage by increasing triacylglycerols and phospholipids. The reduction of steryl esters leads to changes in the lipid particle proteome, including the appearance of new proteins, reflecting flexibility in response to environmental changes [12]. Yeast physiology and development depend on the unsaturation of fatty acids, which affects chain length, and the number of double bonds and their position [11,13]. The unsaturated fatty acid composition in wine yeast may be affected by different factors, such as low temperature, oxygen availability, growth rate, and sterol lipid addition during fermentation [14].

In winemaking, yeast lipid metabolism plays a vital role in modulating fermentation kinetics, preventing sluggish or stuck fermentation, and potentially influencing the production of volatile aroma compounds that shape the sensory quality of wine [15,16]. Supplementation with exogenous unsaturated fatty acids or sterols and targeted manipulation of lipid composition can improve yeast efficiency and alter the volatile composition of wines [17,18]. Conversely, lipid deficiency in yeast can cause sluggishness and lead to incomplete sugar consumption and undesirable byproducts [15]. Moreover, the lipid composition of wine yeast modulates the balance of volatile thiols, higher alcohols, esters, and organic acids, which define wine aroma and flavor [16]. These findings suggest that strain-specific lipidomic analysis not only deepens our understanding of yeast physiology but also provides a practical framework for optimizing industrial fermentation. High-resolution liquid chromatography/mass spectrometry (LC/MS)-based lipidomics is a powerful technique for unraveling the complexity of yeast lipidomes [19]. Advanced lipidomics has enabled the detailed characterization of yeast lipid categories, including fatty acyls (FAs), glycerophospholipids (GPs), sphingolipids (SPs), sterols (STs), and glycerolipids (GLs) [20,21,22]. Recently, untargeted LC/MS and shotgun lipidomics have enabled the comprehensive profiling of hundreds of lipid species with high sensitivity and specificity [23,24]. These approaches have revealed how the wine yeast lipidome is reshaped in response to acetic acid stress [25], copper exposure [26], and protein–lipid microenvironments [27]. Comparative lipidomics further highlights strain-specific lipid signatures that connect metabolic diversity to distinct phenotypes [28].

Despite this progress, significant knowledge gaps remain lacking behind, regarding strain-specific yeast lipidomes and their mechanistic contributions to fermentation outcomes. The systematic lipidomic characterization of diverse wine yeast strains offers insights into how lipid composition governs yeast fitness, fermentation kinetics, and product quality. Therefore, the present study applied high-resolution LC/MS-based comprehensive lipidomic analysis to forty-eight SC wine yeast strains that are isolated in Hokkaido, Japan, to identify and characterize novel and strain-specific lipid signatures that validate physiological performance, underpin stress adaptation, and contribute to the modulation of wine quality.

## 2. Materials and Methods

### 2.1. Materials

Methanol, isopropanol, and chloroform were of LC/MS grade and obtained from Wako Pure Chemical Industries Ltd. (Osaka, Japan). A 1 M ammonium acetate solution was purchased from Sigma-Aldrich (St. Louis, MO, USA). Oleic acid-d9 and the EquiSPLASH^®^ LIPIDOMIX^®^ quantitative standard were procured from Avanti Polar Lipids (Alabaster, AL, USA).

### 2.2. Yeast Samples

*S. cerevisiae* wine yeast strains (48 variants) were used in this study. Strains SC1 to SC48 were isolated from spontaneously fermenting grape musts (pied de cuvee) in a winery in Furano city, Hokkaido, Japan, and deposited in the culture collection of the Research Faculty of Agriculture, Hokkaido University (AHU culture collection). Control is a standard wine starter strain, EC-1118. The yeast cell section follows the previously reported protocols [29]. The strains were revitalized on YM Agar (10 g/L glucose, 5 g/L polypeptone, 3 g/L yeast extract, 3 g/L malt extract and 20 g/L agar) plate at 27 °C for 2 days, followed by preculture in 10 mL YM Broth (same composition as YM Agar except the omission of agar) at 27 °C overnight. Cells were washed with saline and finally resuspended in saline at the concentration of OD_600_ = 1. The precultured cells were inoculated into 10 mL of synthetic grape must (SGM) liquid media at a final concentration of OD_600_ = 0.001 [29]. After the static culture at 27 °C for 14 days, the cells were harvested by centrifugation and stored at −80 °C until the lipidomic analysis was performed. Before analysis, the pellets were resuspended in 0.5 mL Milli-Q water. Four technical replicates (n = 4) of each 100 μL suspension were used for lipid extraction, and the remainder was used to determine protein levels. Each yeast strain was independently cultured, extracted, and analyzed.

### 2.3. Lipid Extraction and Protein Determination of S. cerevisiae Yeast Strains

The 48 wine yeast strains were subjected to total lipid extraction. Lipid extraction was performed using a modified Folch method that was previously optimized in our laboratory [30,31]. The lipid extraction protocol is briefly described as follows: 100 μL of yeast suspension in Milli-Q water was transferred to a 2 mL Eppendorf tube, and 100 μL of methanol was added. The mixture was then homogenized twice for 30 s using a Bead Mill homogenizer (Fisherbrand, Tokyo, Japan). Then, 100 μL of an internal standard (IS) solution mixed in methanol, consisting of oleic acid-d9 (10 μg/mL) and EquiSPLASH (1 μg/mL), was added to the samples. To produce biphasic layers, 400 μL of chloroform was added, and the mixture was thoroughly vortexed for 5 min and centrifuged for 10 min at 15,000 rpm. The lower chloroform layer containing the extracted lipids was transferred to a new 2 mL Eppendorf tube. Following the upper layer, an additional 400 μL of chloroform was added, vortexed for 5 min, and centrifuged for 10 min under the same conditions as earlier. The second extracted chloroform layer was combined with the previous one and evaporated in a vacuum evaporator overnight at 4 °C. The dried lipid extracts were reconstituted in 100 μL of methanol, centrifuged for 10 min, and transferred into LC vials. Then, a 10 μL aliquot of each extract was injected into the LC/MS system. The blank and quality control samples (from pooled yeast extracts) were run between the samples to monitor analytical accuracy, ensuring the data reliability and reproducibility. The protein contents of 48 different SC wine yeast strains were determined according to the protocol described in our previous study [32].

### 2.4. Lipidomic Analysis Using an LC/MS

Lipidomic analysis was performed using a high-performance liquid chromatography (HPLC) system (Shimadzu Corporation, Kyoto, Japan) coupled to an LTQ Orbitrap mass spectrometer (Thermo Fisher Scientific Inc., San Jose, CA, USA). The LC/MS conditions were compatible with our previously published protocol [33,34]. Chromatographic separation was achieved using a reverse-phase Atlantis T3 C18 column (2.1 × 150 mm, 3 µm; Waters, Milford, MA, USA), maintaining the oven temperature at 40 °C. The mobile phase flow rate was 0.2 mL/min. The mobile phase for the LC/MS consisted of A: aqueous 10 mM ammonium acetate (CH_3_COONH_4_) solution, B: isopropanol (IPA), and C: methanol. All MS parameters were identical to those used in our previous study [35]. MS analysis was carried out in both positive and negative electrospray ionization (ESI) modes. The following parameters were maintained: a nitrogen auxiliary gas flow of 20 units, nitrogen sheath gas flow of 50 units, and capillary temperature of 330 °C. The capillary and source voltages were set to 4 kV and 25 V for the positive ionization mode (scan range: *m*/*z* 150–1950), and 3 kV and 10 V for the negative ionization mode (scan range: *m*/*z* 160–1900). The analysis was conducted in ion-trap mode to generate MS/MS spectra in data-dependent acquisition (DDA) mode at a collision energy of 40 V and in Fourier-transform mode with a resolving power of 60,000.

### 2.5. Annotation and Quantification of the Obtained Lipid Metabolites

The raw data acquired from the LC/MS Orbitrap MS were processed using MS-DIAL software (version 4.9) to perform data alignment, peak detection, identification, and mass spectrometry analysis [35]. Xcalibur 2.2 (Thermo Fisher Scientific, Waltham, MA, USA) was used to confirm the peak area integrations and reliably identify and verify the lipid molecular species. By calculating the peak-area ratios of the obtained lipids and the internal standard added to the samples, and then multiplying by the concentration of internal standard added, the relative concentrations in yeast samples were determined. The data were further normalized by the protein concentration in each yeast sample.

### 2.6. Statistical Data Analysis

The acquired and calculated data from both the positive and negative ionization modes were plotted to visualize the statistical analysis using Microsoft Excel 2021 and GraphPad Prism (version 8) software. The data were presented as the mean ± standard error. To gain a deeper understanding of the variations in lipid composition among different yeast strains, multivariate statistical analyses and principal component analysis were performed. Comprehensive data normalization, functional analysis (principal component analysis and clustered heatmaps), and statistical analysis were performed using web-based software, such as MetaboAnalyst (version 5.0). For the functional analysis, the default “none” options were selected for sample normalization, data transformation, and data scaling, as the data had already been normalized by protein amounts. A low-variance filter (interquartile range) and a low-abundance filter (mean intensity value) were applied.

## 3. Results

### 3.1. Multivariate Principal Component Analysis in S. cerevisiae Wine Yeast Variants

**Figure 1:**
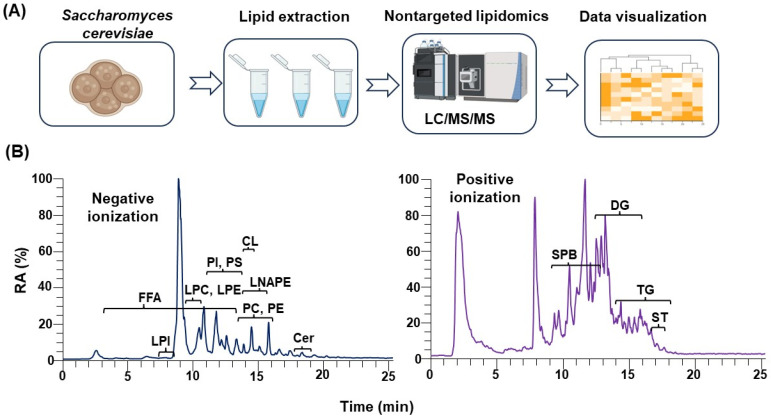
(**A**) Overall study design of *S. cerevisiae* yeast lipidomics. (**B**) Total ion chromatograms of annotated lipids in negative and positive ionization modes. (FFA: free fatty acids, PI: phosphatidylinositol, LPI: lyso-phosphatidylinositol, PC: phosphatidylcholine, LPC: lyso-phosphatidylcholine, PS: phosphatidylserine, PE: phosphatidylethanolamine, LPE: lyso-phosphatidylethanolamine, CL: cardiolipins, Cer: ceramides, LNAPE: N-acyl lysophosphatidylethanolamine, SPB: sphingoid bases, TG: triacylglycerol, DG: diacylglycerol, and ST: sterols).

A comprehensive lipidomic analysis was conducted to identify and characterize the total lipid molecular species, their categories, and relative concentrations across 48 *S. cerevisiae* wine yeast variants. A nontargeted lipidomic approach was employed using high-resolution LC/MS. This analysis enabled the identification of 135 lipid molecular species, classified into five major lipid categories and 15 lipid subclasses, in the 48 wine yeast variants. A list of the identified lipid molecular species and their relative concentrations across yeast samples is provided in the Supporting Information (Table S1). The study design for the wine yeast lipidome analysis is shown in Figure 1A. The results of total ion chromatographs (TIC) in both negative and positive ionization modes, showing diverse lipid subclasses, are shown in Figure 1B. The TIC in negative mode contains the lipid subclasses, including fatty acyls (FA), phosphatidylcholine (PC), phosphatidylethanolamine (PE), phosphatidylinositol (PI), phosphatidylserine (PS), cardiolipins (CL), lysophosphatidylcholine (LPC), lysophosphatidylethanolamine (LPE), N-acyl lysophosphatidylethanolamine (LNAPE), lysophosphatidylinositol (LPI), and ceramides (Cer). Positive-mode TIC included STs, sphingoid bases (SPB), diacylglycerols (DGs), and triacylglycerols (TGs).

**Figure 2:**
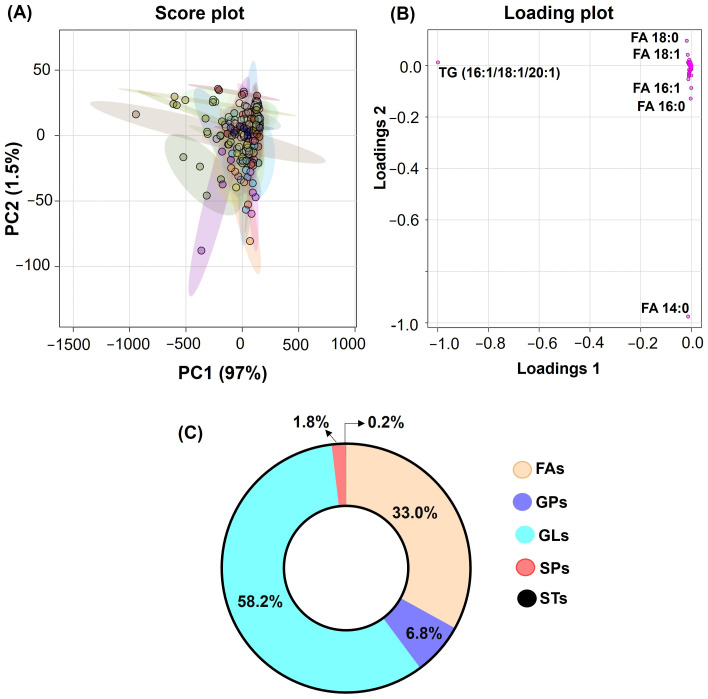
(**A**) Principal component analysis (PCA) score plot showing the distribution of samples across 48 *S. cerevisiae* strains. (**B**) Loading plot corresponds to PCA. Statistical significance of group separation was evaluated using permutational multivariate analysis of variance (PERMANOVA). Distances were calculated using Euclidean distance based on the principal components displayed in the plot. For comparisons of multiple groups, both overall and pairwise PERMANOVA results were assessed (F-value: 6.2811; R-squared: 0.6722; *p*-value: 0.001). (**C**) Percentage distribution of five major lipid classes characterized in *S. cerevisiae*. FA: fatty acyls, GPs: Glycerophospholipids, GLs: Glycerolipids, SPs: Sphingolipids, ST: Sterols.

Multivariate principal component analysis (PCA) revealed a group separation among the 48 SC wine yeast variants, with PC1 and PC2 explaining 97% and 1.5% of the variance, respectively, as shown in Figure 2A. The lipid species that contributed most strongly to the variance were TG (16:1/18:1/20:1), FA18:0, FA 18:1, FA 16:0, and FA 16:1, as shown in the PCA loading plot (Figure 2B). The observed PCA group separation of analyzed samples is likely attributable to several factors: dominance of a small number of highly abundant lipid species that disproportionately drive variance, the specific normalization and scaling procedures applied before analysis, and technical variability or batch effects introduced during sample preparation and LC/MS acquisition. Variations in lipid extraction efficiency, differential ionization yield across molecular species, and inherent biological heterogeneity between yeast strains may further influence clustering and the variance captured by PC1 and PC2. Illustrating the proportion of lipid percentages, Figure 2C represents the five major lipid categories obtained in the yeast samples, indicating that GLs had the highest abundance (58.2%), followed by fatty acyls (33.0%) and GPs (6.8%).

### 3.2. Analysis of Free Fatty Acid Compositions

**Figure 3:**
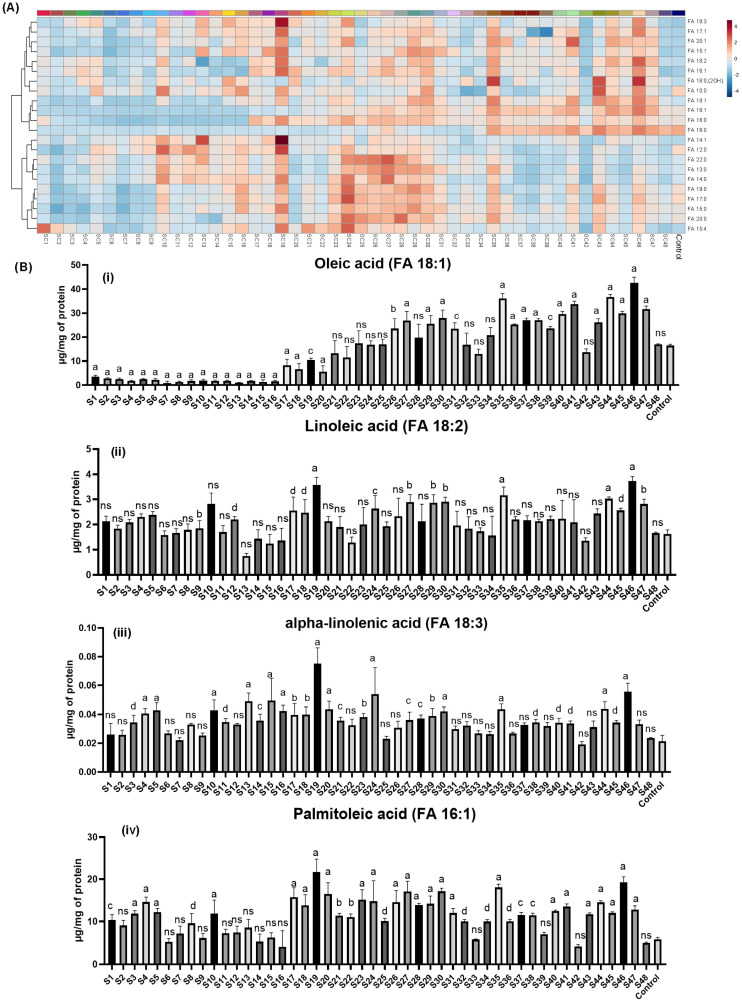
(**A**). Clustered heatmap analysis of the identified fatty acid lipid species in S. cerevisiae strains. The heatmap was generated based on the Euclidean distance measure and the Ward clustering algorithm using different lipid class-specific analyses. (**B**) Relative concentrations of selected fatty acids expressed in μg/mg of protein. (**i**) oleic acid (FA 18:1), (**ii**) linoleic acid (FA 18:2), (**iii**) α-linolenic acid (FA 18:3), and (**iv**) palmitoleic acid (FA 16:1). (S1 in the figure refers to SC1, with the same correspondence applying to other labels (S2 → SC2, S3 → SC3, etc.)). Ordinary One-Way ANOVA with Dunnett multiple comparison test was applied (comparisons were made with control. a: *p* < 0.0001, b: *p* < 0.001, c: *p* < 0.01, d: *p* < 0.05 and ns: non-significant).

Untargeted analysis of 48 *S. cerevisiae* wine yeast variants revealed 22 free fatty acids, including palmitoleic, oleic, linoleic, and alpha-linolenic acids, which are known to influence wine quality during fermentation. Fatty acids are key regulators of yeast stress during fermentation process. The Z-score normalized clustered heatmap of the obtained fatty acids showed strong variation among the 48 yeast strains, with high concentrations of SC19, SC24, SC30, SC36, and SC46 variants, while SC2, SC6, and SC7 had the lowest values, as shown in Figure 3A. An intense red color represents a high concentration of lipid species; conversely, an intense shade of blue represents a lower concentration. The individual values of the saturated fatty acids (SFAs), monounsaturated fatty acids (MUFAs), and polyunsaturated fatty acids (PUFAs) of the 48 SC wine yeast strains are given in Table 1, showing the mean value ± standard error. Samples (*n* = 4) were used for lipidomic analyses. SFAs were present at higher levels than MUFAs and PUFAs. The SC27 strain showed the highest amount of SFAs (188.30 ± 16.14), followed by the SC24 (180.38 ± 22.15) and SC19 (178.87 ± 13.59) variants. In contrast, the SC2 variant had the lowest value of 83.86 ± 3.87 among all the yeast strains. Oleic acid (FA 18:1) exhibited strain-specific variation with the SC46 variant, which contained the highest levels among the other variants. In general, SC1–16 contained relatively low concentrations, whereas strains SC 17–48 displayed higher concentrations, as shown in Figure 3B(i). For example, the SC46 variant showed almost 43-fold higher levels of oleic acid content than the SC13 variant. A similar pattern of variation was observed for linoleic acid (FA 18:2), alpha-linolenic acid (FA 18:3) and palmitoleic acid (FA 16:1) among the 48 yeast variants (Figure 3B(ii–iv)). Linoleic acid (ω-6) is almost 5-fold higher in SC19 and SC47 strains compared to the SC13 variant. The alpha-linolenic acid showed the highest levels in the SC19 strain but the lowest in the SC42 variant. Palmitoleic acid showed similar trends as that of alpha-linolenic acid across the yeast variants. Oleic acid and palmitoleic acid appear to be the most predominant MUFAs. Overall, this result suggests large variations in free fatty acid levels between each *S. cerevisiae* yeast strain.

### 3.3. Clustered Heatmap Analysis of Other Complex Lipidomes in Yeast

**Figure 4:**
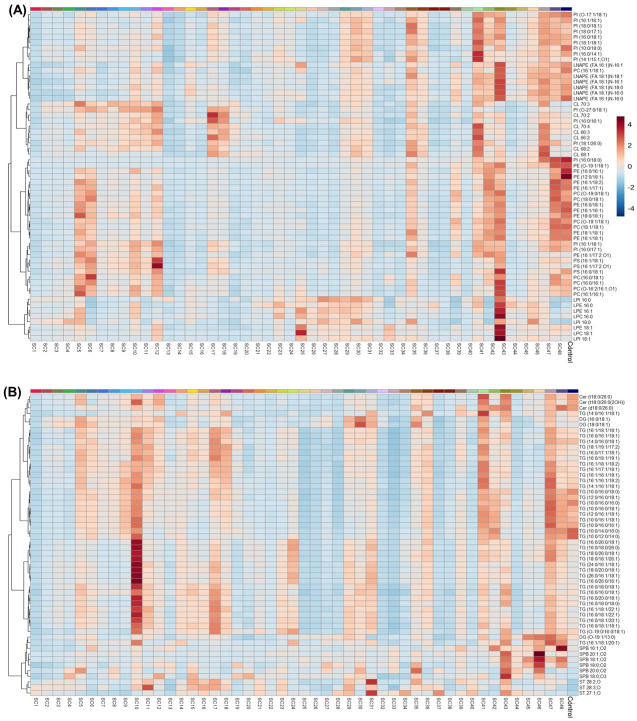
Clustered heatmap visualization of relative concentrations of lipids in S. cerevisiae strains. (**A**). Glycerophospholipids. (**B**). Glycerolipids, sphingolipids, and sterols. The intense red color represents a high concentration of lipid species, whereas an intense blue color represents a lower concentration. The heatmap was generated based on the Euclidean distance measure and the Ward clustering algorithm using different lipid class-specific analyses.

Clustered heatmap analysis was performed to evaluate the distributions of the glycerophospholipids (GPs), glycerolipids (GLs), sphingolipids (SPs), and sterols (STs) compositions (Figure 4A,B) across the 48 wine yeast strains. The heatmap of GPs (Figure 4A) shows substantial concentration variations among the various yeast strains. Lysophospholipids were present at relatively higher levels in SC43 and SC25 compared to other strains. PIs and PEs showed relatively higher concentrations in SC41 and SC48, respectively. The *S. cerevisiae* variants SC41, SC43 and SC48 strains displayed a high intensity with red color, indicating elevated levels of multiple GP subclasses. Conversely, yeast strains SC1, SC13, SC14, and several other variants exhibited low concentrations, as reflected by the blue color. Novel bioactive lipids such as N-acyl lysophosphatidylethanolamines (LNAPEs) were also detected at higher concentrations in the SC43 strain, suggesting potential applications in the wine industry. The combined heatmap of GLs, STs, and SPs (Figure 4B) highlights prominent changes in lipid concentrations across *S. cerevisiae* strains. Among GLs, TGs were the most predominant species and were abundant in SC10, SC41, and SC47 strains. These TGs were acylated with saturated and monounsaturated fatty acids. Very few molecular species of STs and SPs were detected. Among SPs, sphingoid bases occurred at relatively higher levels in SC46 and SC43 compared to other strains. Among all lipid species visualized in Figure 4B, GLs were the most abundant, followed by SPs and STs, underscoring their dominant role in the yeast lipidome.

### 3.4. Identification and Characterization of N-Acyl Lysophosphatidylethanolamine in Yeast Samples

**Figure 5:**
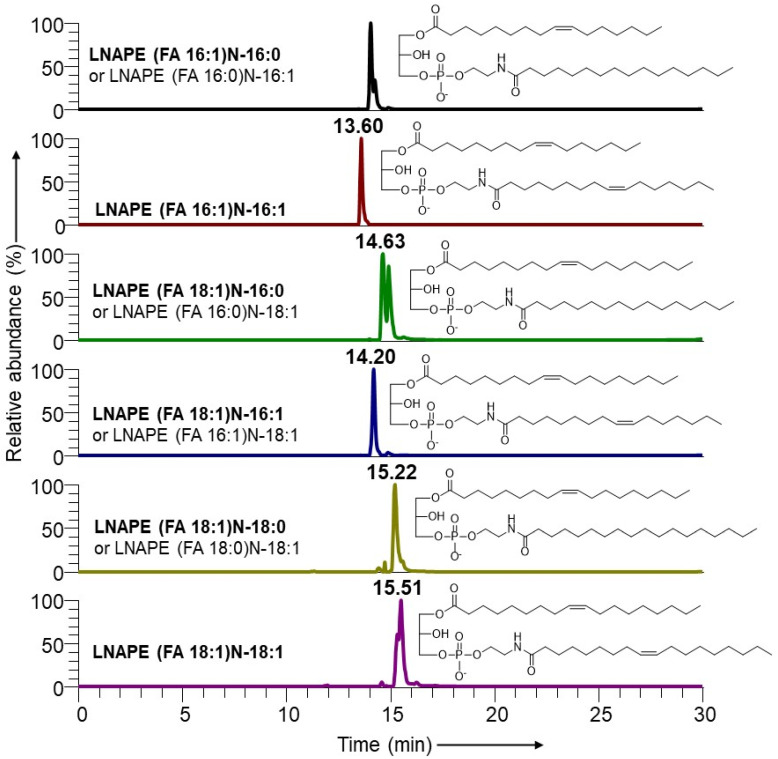
Extracted ion chromatograms of the identified LNAPEs and their putative structures characterized in S. cerevisiae strains.

A comprehensive lipidomic analysis of 48 strains of *S. cerevisiae* led to the identification and characterization of six lipid molecular species of N-acyl lysophosphatidylethanolamine (LNAPE), a subclass of GPs. The identified and characterized species are LNAPE (FA 16:1)N-16:0 (or LNAPE (16:0)N-16:1), LNAPE (FA 16:1)N-16:1, LNAPE (FA 18:1)N-16:0 (or LNAPE (FA 16:0)N-18:1), LNAPE (FA 18:1)N-16:1 (or LNAPE (FA 16:1)N-18:1), LNAPE (FA 18:1)N-18:0 (or LNAPE (FA 18:0)N-18:1), and LNAPE (FA 18:1)N-18:1. The extracted ion chromatograms of all identified LNAPEs are shown in Figure 5. All the LNAPEs eluted at retention times between 13 and 15 min, generating [M-H]^−^ ion, as the precursor ion in the negative ionization mode of LC/MS. These LNAPEs were putatively identified based on their retention times, high-resolution mass spectra, and MS/MS fragmentation.

**Figure 6:**
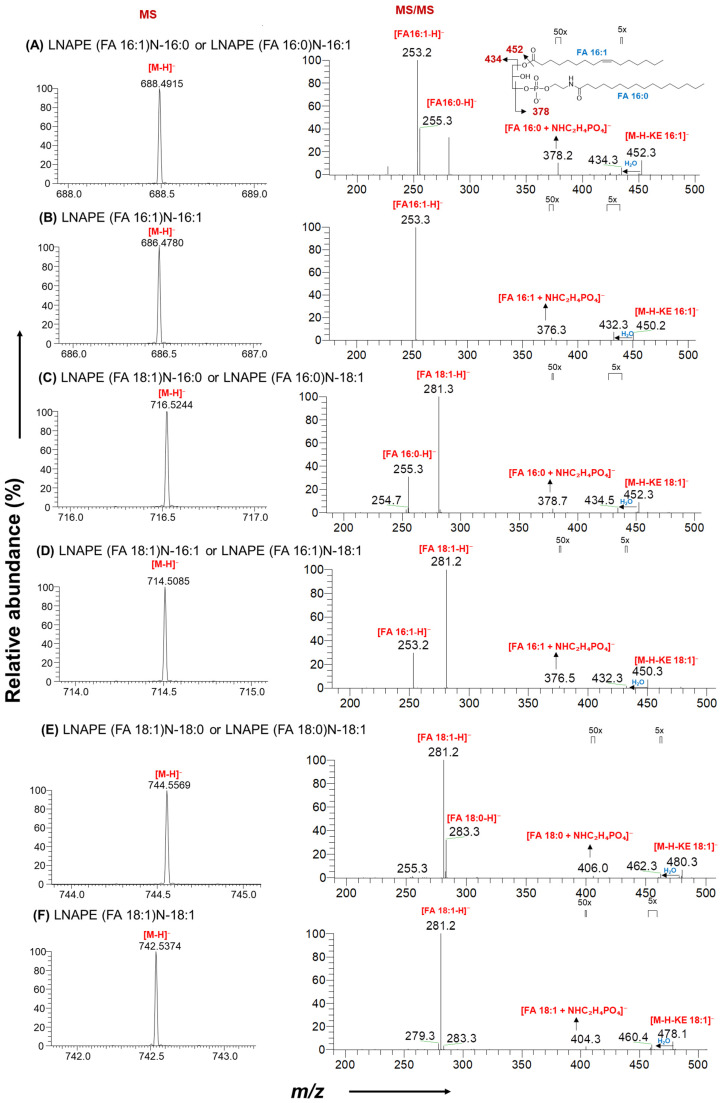
Representative mass spectra (MS) and tandem mass spectra (MS/MS) of N-acyl lysophosphatidylethanolamine (LNAPEs) identified and characterized in *S. cerevisiae* (**A**–**F**). The spectra display the precursor ions and characteristic fragment ions used for structural elucidation and annotation of LNAPE lipid molecular species.

The MS spectra and MS/MS fragmentation patterns of the detected LNAPEs are shown in Figure 6. The lipid metabolite eluted at a retention time (RT) of 14.05 min and ionized to generate the [M-H]^−^ ion with an experimental *m*/*z* of 688.4915 (theoretical *m*/*z* of 688.4923, mass error: −1.16 ppm). The MS/MS spectra showed an *m*/*z* of 452.3, indicating the loss of FA 16:1 as ketene (KE), followed by a neutral loss of water, giving *m*/*z* = 434.3. The loss of [FA 16:1-H]^−^ and [FA 16:0-H]^−^ produced fragment ions at *m*/*z* 253.2 and 255.3 respectively, indicating the presence of FA 16:1 and FA 16:0. These findings further confirm that the identified compound is a mixture of two regioisomeric LNAPEs. Furthermore, the loss of 312 from the parent ion to give a fragment ion at *m*/*z* of 378.2, which is described as [FA 16:0 + NHC_2_H_4_PO_4_]^−^, collectively supports the putative identification of an isomeric mixture of LNAPE (FA 16:1) N-16:0 and LNAPE (FA16:0) N-16:1, as shown in Figure 6A. The relative abundance of the fatty acid fragments at *m*/*z* 253 and 255 determines the ratio of these isomers. As the fragment at *m*/*z* 253 is more abundant than that at *m*/*z* 255, the predominant isomer is likely LNAPE (FA 16:1) N-16:0. The lipid molecule eluted at 13.60 min with an [M-H]^−^ ion, with an experimental *m*/*z* of 686.4780 (theoretical *m*/*z*: 686.4766, mass error: −2.03 ppm). The MS/MS spectrum showed an *m*/*z* of 450.2, with a loss of FA 16:1 as KE, followed by a neutral loss of water, giving *m*/*z* of 432.3. The loss of [FA 16:1-H]^−^ produced a fragment ion at *m*/*z* = 253.3, and the loss of 310 from the parent ion to give a fragment ion at *m*/*z* of 376.5, which is described as [FA 16:1 + NHC_2_H_4_PO_4_]^−^, which putatively identifies the presence of LNAPE (FA 16:1)N-16:1, as visualized in Figure 6B.

The molecule eluted at 14.63 min, with an experimental *m*/*z* of 716.5244 (theoretical *m*/*z*: 716.5236, mass error: 1.11 ppm), yielding an [M-H]^−^ ion. The MS/MS spectrum generated an *m*/*z* of 452.3, showing the loss of FA 18:1 as KE, followed by a neutral loss of water, giving *m*/*z* of 434.3. The loss of [FA 18:1-H]^−^ and [FA 16:0-H]^−^ produced fragment ions at *m*/*z* 281.3 and 255.3 respectively, indicating the presence of FA 18:1 and FA 16:0. These findings further confirm that the identified compound is a mixture of two regioisomeric LNAPEs. Furthermore, the loss of 338 from the parent ion to give a fragment ion at *m*/*z* of 378.7, which is described as [FA 16:0 + NHC_2_H_4_PO_4_]^−^, collectively supports the putative identification of an isomeric mixture of LNAPE (FA 18:1)N-16:0 and LNAPE (FA 16:0)N-18:1 (Figure 6C). The relative abundance of the fatty acid fragments at *m*/*z* 255 and 281 determines the ratio of these isomers. As the fragment at *m*/*z* 281 is more abundant than that at *m*/*z* 255, the predominant isomer is likely LNAPE (FA 18:1) N-16:0.

Similarly, the lipid species at RT 14.20 min exhibited an experimental *m*/*z* of 714.5085 (theoretical *m*/*z*: 714.5079, mass error: 0.83 ppm) and in the MS/MS spectra, loss of FA 18:1 as KE, followed by a neutral loss of water, giving ions at *m*/*z* 450.3 and 432.2, respectively. The loss of [FA 18:1-H]^−^ and [FA 16:1-H]^−^ produced fragment ions at *m*/*z* 281.3 and 253.2 respectively, indicating the presence of FA 18:1 and FA 16:1. These findings further confirm that the identified compound is a mixture of two regioisomeric LNAPEs. Furthermore, the loss of 338 from the parent ion to give a fragment ion at *m*/*z* of 376.5, which is described as [FA 16:1 + NHC_2_H_4_PO_4_]^−^, collectively supports the putative identification of an isomeric mixture of LNAPE (FA 18:1)N-16:1 and LNAPE (FA 16:1)N-18:1 (Figure 6D). The relative abundance of the fatty acid fragments at *m*/*z* 253 and 281 determines the ratio of these isomers. As the fragment at *m*/*z* 281 is more abundant than that at *m*/*z* 253, the predominant isomer is likely LNAPE (FA 18:1) N-16:1.

The lipid metabolites eluted at RT 15.22 min, with an [M-H]^−^ ion and experimental *m*/*z* of 744.5569 (theoretical *m*/*z*: 744.5549, mass error: 2.68 ppm), produced fragment ions with *m*/*z* values of 450.3 and 432.2 in response to [M-H-KE 18:1]^−^ and neutral water loss in its MS/MS spectrum, respectively. The loss of [FA 18:1-H]^−^ and [FA 18:0-H]^−^ produced fragment ions at *m*/*z* 281.3 and 283.3 respectively, indicating the presence of FA 18:1 and FA 18:0. These findings further confirm that the identified compound is a mixture of two regioisomeric LNAPEs. Furthermore, the loss of 338 from the parent ion to give a fragment ion at *m*/*z* of 406.0, which is described as [FA 18:0 + NHC_2_H_4_PO_4_]^−^, collectively supports the putative identification of an isomeric mixture of LNAPE (FA 18:1)N-18:0 and LNAPE (FA 18:1)N-18:1, as shown in Figure 6E. The relative abundance of the fatty acid fragments at *m*/*z* 281 and 283 determines the ratio of these isomers. As the fragment at *m*/*z* 281 is more abundant than that at *m*/*z* 283, the predominant isomer is likely LNAPE (FA 18:1) N-18:0. Finally, the lipid molecule at the RT of 15.51 min, with an experimental *m*/*z* of 742.5374 (theoretical *m*/*z*: 742.5392, mass error: −2.42 ppm), generated [M-H]^−^ as its precursor ion. In the MS/MS spectrum, the [M-H-KE 18:1]^−^ ion gives *m*/*z* = 478.3, and losing the neutral water molecule produced a fragment ion at *m*/*z* of 460.3. The loss of [FA 18:1-H]^−^ produced a fragment ion at *m*/*z* = 281.2, and the loss of 338 from the parent ion to give a fragment ion at *m*/*z* of 404.5, which is described as [FA 18:1 + NHC_2_H_4_PO_4_]^−^, putatively identifies the presence of LNAPE (FA 18:1)N-18:1, as shown in Figure 6F. Although these lipids demonstrated potential anti-inflammatory activities in murine models, their benefits for yeast metabolism or winemaking remain undetermined.

## 4. Discussion

A comprehensive lipid analysis of 48 *S. cerevisiae* yeast strains enables us to determine the detailed lipid profiles and identify novel lipids and their potential contributions to the food and wine industries. *S. cerevisiae* metabolites play a crucial role in shaping the taste, flavor, and texture of foods and drinks during fermentation, particularly in the wine industry [36]. The study identified a total of 135 lipid species, encompassing five major lipid classes and 15 lipid subclasses across 48 yeast variants, using nontargeted high-resolution LC/MS. Previous studies have reported more structural diversity of lipid molecular species in yeast extracts using MS-based shotgun lipidome and LC/MS analysis [19,22]. However, these were limited to very few strains, and LNAPEs were unreported.

In this study, we identified 22 free fatty acids ranging from C10 to C22 in chain length. Most of the fatty acids detected were SFAs and MUFAs. The abundance of oleic acid and palmitoleic acid in *S. cerevisiae* are consistent with previous reports [37]. Long-chain saturated fatty acids are known to contribute to membrane rigidity and maintain structural integrity under stress, aiding yeast survival [38]. In *S. cerevisiae,* it is reported that fatty acid chain length may increase with increased temperature and uncommon fatty acids are used as biomarkers to monitor metabolic changes during fermentation [38,39]. Furthermore, the addition of unsaturated fatty acids, including linoleic and α-linolenic acid, influences fermentation kinetics in the wine and food industries by changing taste, flavor, and color stability [40,41,42]. We identified multiple subclasses of GPs, including PC, PE, PI, PS, LPE, LPI, and CL, which are highly abundant in the yeast lipidome. Previous studies have reported the presence of over 800 lipid species in *S. cerevisiae* [10,43]. In our study, we detected 135 lipid molecular species. This difference may be attributed to methodological limitations (including instrument sensitivity and mode of data acquisition), strain specificity, and variations in culture conditions, all of which can influence lipid profiles. GPs play a vital role in the yeast lipidome, as they influence physiological and metabolic pathways essential for efficient fermentation and function as fundamental structural components of the cell membrane [28,43].

Among GLs identified in our analysis, the TGs were the most abundant subclass. High TG accumulation levels are known to affect exponential yeast growth under adverse conditions and fermentation mechanisms [44]. GLs play significant roles in cell signaling, membrane structure, trafficking, and anchoring of membrane proteins [43]. TG biosynthesis is regulated by complicated metabolic networks in response to dietary requirements and yeast development [45]. Furthermore, the STs and SPs identified in this analysis were found to contribute to the fermentation kinetics in yeast lipidomics in the food industry [37,46]. The role of STs, particularly ergosterol produced under aerobic conditions, and plant sterols generated in an anaerobic environment, is essential for maintaining yeast cell viability during wine fermentation under adverse conditions, such as ethanol stress, preventing stuck fermentation [46]. During fermentation, SC remodels its membrane, showing decreased cell diameter and integrity, as well as increased ergosterol and oleic acid. It can alter phospholipid levels and gene expression to enhance membrane fluidity and may contribute to improving ethanol tolerance [47]. SC, a cornerstone of industrial microbiology used in food fermentations, faces osmotic stress from high sugar or salt loads via osmolyte accumulation, which alters the lipid metabolic pathways, and plays a crucial role in stress-regulation, adaptive evolution, and naturally osmo-tolerant strains that together offer routes to improve industrial fermentations [48]. Ethanol yield optimization is significant to maintain efficient production of fuel alcohol and alcoholic beverages, but rising ethanol concentrations during fermentation can hamper cellular function, which leads to stalled or sluggish conversion of sugars to ethanol [8].

Notably, we detected several LNAPEs, a subclass of GPs that may play a significant role in yeast lipid metabolism. In previous studies, LNAPEs were identified and characterized in various plant and food samples, including herbal teas, food grains, and fish [34,49,50]. Previously, quantitative shotgun mass spectrometry quantified 250 lipid species with 21 lipid classes covering 95% of the yeast lipidome [22]. The major LPE acyltransferase in *S. cerevisiae* has been identified and characterized [51]. The first identification of N-acylethanolamines (NAEs) and N-acyl phosphatidylethanolamines (NAPEs) in wild-type SC was reported by Merkel et al., and they demonstrated that oxidative stress can significantly increase the NAE levels [52]. However, NAPEs lysoforms, i.e., LNAPEs, remain uncharacterized. Bioactive NAEs are produced from membrane phospholipids via NAPE and formed through N-acyltransferase activity in living cells, supporting their role in NAE biosynthesis as reported [53]. Acyl chain remodeling of glycerophospholipids plays an important role in membrane dynamics in yeast [54]. Strain-specific yeast lipidomes can modulate fermentation efficiency to alter the taste, flavor, color, and texture of wine [55,56]. However, this study has certain limitations. The lipid analysis was semi-quantitative, which may restrict the precision of comparative assessments across strains. Additionally, the collection time and origin of the various *S. cerevisiae* strains were not fully controlled, potentially influencing their lipid composition. Although all strains were cultivated under standardized laboratory conditions, lipid biosynthesis may still vary depending on the environmental and agronomic factors of the original isolation sites. This study demonstrates the variation in fatty acid compositions, various strain-based comparisons among the analyzed yeast strains and focuses on the putative identification of novel bioactive lipids, such as LNAPEs, based on their retention time behavior, accurate mass measurements, and MS/MS fragmentation patterns obtained in an untargeted LC/MS study. Furthermore, this study may advance the validation of future functional studies, including fermentation, ethanol production, aroma profile analysis, residual sugar measurement, and stress-tolerance evaluation in wine yeast for potential wine quality development.

## 5. Conclusions

This study presents a comprehensive lipidomic analysis of 48 *Saccharomyces cerevisiae* strains using a nontargeted LC/MS approach. In total, 135 lipid molecular species were identified and characterized, encompassing 15 subclasses across five major lipid classes. Notably, strains SC19 and SC47 exhibited relatively high concentrations of fatty acids such as oleic, linoleic, and α-linolenic acids. Strains SC43, SC47, and SC48 exhibited higher relative lipid concentrations than the other strains. Importantly, six bioactive lipids, including LNAPEs, were putatively identified and structurally characterized based on high-resolution MS and MS/MS fragmentation patterns. These findings suggest the physiological and metabolic relevance of these lipids in yeast. Overall, this study improves our understanding of strain-specific lipidomic diversity in *S. cerevisiae* and may inform future efforts to optimize fermentation processes and enhance the sensory quality of foods and beverages.

## Figures and Tables

**Table 1:** Amount (µg/mg of protein) of saturated fatty acids (SFAs), monounsaturated fatty acids (MUFAs), and polyunsaturated fatty acids (PUFAs) characterized in *S. cerevisiae* wine yeast strains (Data represented as mean ± standard error (SE), *n* = 4).

SC	Strain No.	SFAs (Mean ± SE)	MUFAs (Mean ± SE)	PUFAs (Mean ± SE)
SC_1	AHU 5099	109.63 ± 7.86	15.56 ± 0.97	2.22 ± 0.11
SC_2	AHU 5100	83.86 ± 3.87	13.26 ± 0.56	1.91 ± 0.07
SC_3	AHU 5101	99.27 ± 2.44	15.98 ± 0.44	2.15 ± 0.06
SC_4	AHU 5102	98.17 ± 2.57	17.99 ± 0.61	2.37 ± 0.06
SC_5	AHU 5103	111.83 ± 2.79	16.44 ± 0.32	2.46 ± 0.06
SC_6	AHU 5104	97.85 ± 6.49	8.651 ± 0.63	1.63 ± 0.08
SC_7	AHU 5105	86.45 ± 4.48	9.555 ± 1.23	1.71 ± 0.08
SC_8	AHU 5106	100.02 ± 0.58	12.67 ± 1.17	1.84 ± 0.12
SC_9	AHU 5107	104.82 ± 1.20	9.612 ± 0.66	1.91 ± 0.14
SC_10	AHU 5108	148.20 ± 6.45	16.39 ± 1.77	2.91 ± 0.21
SC_11	AHU 5109	113.71 ± 1.31	10.91 ± 0.26	1.77 ± 0.12
SC_12	AHU 5110	128.44 ± 1.59	11.01 ± 0.92	2.26 ± 0.06
SC_13	AHU 5111	121.98 ± 2.78	12.18 ± 1.05	0.83 ± 0.04
SC_14	AHU 5112	115.96 ± 9.06	8.935 ± 0.91	1.51 ± 0.18
SC_15	AHU 5113	122.90 ± 16.70	9.820 ± 0.79	1.34 ± 0.19
SC_16	AHU 5114	125.90 ± 8.70	7.963 ± 2.07	1.46 ± 0.23
SC_17	AHU 5115	141.65 ± 12.06	26.07 ± 2.10	2.63 ± 0.27
SC_18	AHU 5116	130.01 ± 12.20	22.58 ± 1.80	2.54 ± 0.26
SC_19	AHU 5117	178.87 ± 13.59	35.66 ± 1.80	3.71 ± 0.16
SC_20	AHU 5118	110.60 ± 3.62	23.91 ± 2.08	2.21 ± 0.09
SC_21	AHU 5119	116.87 ± 3.99	26.63 ± 2.91	2.00 ± 0.21
SC_22	AHU 5120	107.52 ± 3.15	24.30 ± 2.45	1.37 ± 0.10
SC_23	AHU 5121	146.01 ± 7.45	34.70 ± 3.78	2.10 ± 0.32
SC_24	AHU 5122	180.38 ± 22.15	33.89 ± 2.67	2.76 ± 0.23
SC_25	AHU 5123	136.08 ± 5.61	28.59 ± 0.83	2.01 ± 0.08
SC_26	AHU 5124	162.29 ± 13.77	40.18 ± 3.23	2.40 ± 0.36
SC_27	AHU 5125	188.30 ± 16.14	46.12 ± 2.99	2.98 ± 0.15
SC_28	AHU 5126	147.37 ± 3.08	35.89 ± 2.76	2.22 ± 0.33
SC_29	AHU 5127	150.82 ± 8.80	42.23 ± 1.45	2.94 ± 0.16
SC_30	AHU 5128	143.19 ± 4.75	47.46 ± 2.01	2.99 ± 0.08
SC_31	AHU 5129	119.54 ± 3.53	37.49 ± 1.78	2.03 ± 0.28
SC_32	AHU 5130	101.72 ± 3.85	28.54 ± 2.43	1.90 ± 0.24
SC_33	AHU 5131	107.16 ± 1.82	20.13 ± 1.04	1.79 ± 0.07
SC_34	AHU 5132	110.74 ± 6.02	32.61 ± 1.74	1.62 ± 0.38
SC_35	AHU 5133	165.44 ± 7.50	56.79 ± 1.30	3.25 ± 0.16
SC_36	AHU 5134	127.80 ± 3.84	37.20 ± 0.24	2.26 ± 0.06
SC_37	AHU 5135	111.36 ± 5.55	40.35 ± 0.79	2.23 ± 0.09
SC_38	AHU 5136	106.89 ± 2.32	39.65 ± 0.36	2.19 ± 0.04
SC_39	AHU 5137	121.61 ± 1.65	31.54 ± 0.43	2.28 ± 0.05
SC_40	AHU 5138	128.32 ± 7.02	44.20 ± 0.63	2.29 ± 0.36
SC_41	AHU 5139	144.46 ± 2.31	49.82 ± 1.00	2.16 ± 0.44
SC_42	AHU 5140	96.11 ± 0.57	19.05 ± 0.91	1.40 ± 0.05
SC_43	AHU 5141	157.30 ± 4.93	39.63 ± 0.99	2.52 ± 0.09
SC_44	AHU 5142	131.49 ± 5.18	53.77 ± 0.54	3.10 ± 0.03
SC_45	AHU 5143	117.03 ± 2.39	44.09 ± 0.50	2.62 ± 0.04
SC_46	AHU 5144	161.70 ± 1.72	64.69 ± 1.77	3.83 ± 0.09
SC_47	AHU 5145	132.17 ± 3.55	46.76 ± 1.08	2.88 ± 0.09
SC_48	AHU 5146	114.53 ± 1.02	23.32 ± 0.05	1.71 ± 0.01
	Control (EC1118) (AHU4636)	133.15 ± 3.52	23.62 ± 0.52	1.67 ± 0.08

## Data Availability

The original contributions presented in this study are included in the article/Supplementary Material. Further inquiries can be directed to the corresponding authors.

## Supplementary Materials

The following supporting information can be downloaded at: https://www.mdpi.com/article/10.3390/microorganisms14061260/s1, Table S1: List of identified lipid metabolites from 48 *Saccharomyces cerevisiae* strains and their relative concentrations (µg/mg).

## Abbreviations

The following abbreviations are used in this manuscript:

SC	*Saccharomyces cerevisiae*
LC/MS	Liquid Chromatography Mass Spectrometry
LNAPE	N-acyl Lysophosphatidylethanolamines
SFA	Saturated Fatty Acid
MUFA	Monounsaturated Fatty Acid
PUFA	Polyunsaturated Fatty Acid.
TIC	Total Ion Chromatogram
DDA	Data-Dependent Acquisition
AHU	Faculty of Agriculture, Hokkaido University
SGM	Synthetic Grape Must
FAs	Fatty Acyls
GPs	Glycerophospholipids
GLs	Glycerolipids
STs	Sterols
SPs	Sphingolipids
NAPE	N-acyl Phosphatidylethanolamine
QC	Quality Control
*m*/*z*	Mass to Charge Ratio
MS	Mass Spectrometry
MS/MS	Tandem Mass Spectrometry
LPC	Lysophosphatidylcholine
LPE	Lysophosphatidylethanolamine
LPI	Lysophosphatidylinositol
TG	Triacylglycerol
PC	Phosphatidylcholine
PI	Phosphatidylinositol
PE	Phosphatidylethanolamine
PS	Phosphatidylserine
CL	Cardiolipin
FFA	Free Fatty Acids
RT	Retention Time
Cer	Ceramides
SE	Steryl Ester
DG	Diacylglycerols
SPB	Sphingoid Base
ESI	Electrospray Ionization
IPA	Isopropyl Alcohol
kV	Kilovolt
rpm	Revolutions Per Minute
OD_600_	Optical Density at 600 nm
PCA	Principal Component Analysis
EICs	Extracted Ion Chromatograms

## References

[1] Renne M.F., Bachmann R., Klose C., Hentrich T., Schulze-Hentrich J.M., Ernst R. (2025). Laboratory Yeast Strains Are Highly Diverse in Lipid Metabolism. bioRxiv.

[2] Rodríguez-Vargas S., Sánchez-García A., Martínez-Rivas J.M., Prieto J.A., Randez-Gil F. (2007). Fluidization of Membrane Lipids Enhances the Tolerance of *Saccharomyces Cerevisiae* to Freezing and Salt Stress. Appl. Environ. Microbiol..

[3] Wolf I.R., Marques L.F., de Almeida L.F., Lázari L.C., de Moraes L.N., Cardoso L.H., Alves C.C.d.O., Nakajima R.T., Schnepper A.P., Golim M.d.A. (2023). Integrative Analysis of the Ethanol Tolerance of *Saccharomyces cerevisiae*. Int. J. Mol. Sci..

[4] Henderson C.M., Block D.E. (2014). Examining the Role of Membrane Lipid Composition in Determining the Ethanol Tolerance of *Saccharomyces cerevisiae*. Appl. Environ. Microbiol..

[5] Lairón-Peris M., Routledge S.J., Linney J.A., Alonso-del-Real J., Spickett C.M., Pitt A.R., Guillamón J.M., Barrio E., Goddard A.D., Querol A. (2021). Lipid Composition Analysis Reveals Mechanisms of Ethanol Tolerance in the Model Yeast *Saccharomyces cerevisiae*. Appl. Environ. Microbiol..

[6] Botstein D., Fink G.R. (2011). Yeast: An Experimental Organism for 21st Century Biology. Genetics.

[7] Berthels N.J., Cordero Otero R.R., Bauer F.F., Thevelein J.M., Pretorius I.S. (2004). Discrepancy in Glucose and Fructose Utilisation during Fermentation by *Saccharomyces cerevisiae* Wine Yeast Strains. FEMS Yeast Res..

[8] Henderson C.M., Lozada-Contreras M., Jiranek V., Longo M.L., Block D.E. (2013). Ethanol Production and Maximum Cell Growth Are Highly Correlated with Membrane Lipid Composition during Fermentation as Determined by Lipidomic Analysis of 22 *Saccharomyces cerevisiae* Strains. Appl. Environ. Microbiol..

[9] Veen M., Lang C. (2004). Production of Lipid Compounds in the Yeast *Saccharomyces cerevisiae*. Appl. Microbiol. Biotechnol..

[10] Danne-Rasche N., Rubenzucker S., Ahrends R. (2020). Uncovering the Complexity of the Yeast Lipidome by Means of NLC/NSI-MS/MS. Anal. Chim. Acta.

[11] Prasad R. (1985). Lipids in the Structure and Function of Yeast Membrane. Adv. Lipid Res..

[12] Grillitsch K., Connerth M., Köfeler H., Arrey T.N., Rietschel B., Wagner B., Karas M., Daum G. (2011). Lipid Particles/Droplets of the Yeast *Saccharomyces cerevisiae* Revisited: Lipidome Meets Proteome. Biochim. Biophys. Acta (BBA)—Mol. Cell Biol. Lipids.

[13] Guilloux-Benatier M., Le Fur Y., Feuillat M. (1998). Influence of Fatty Acids on the Growth of Wine Microorganisms *Saccharomyces cerevisiae* and *Oenococcus oeni*. J. Ind. Microbiol. Biotechnol..

[14] Tronchoni J., Rozès N., Querol A., Guillamón J.M. (2012). Lipid Composition of Wine Strains of *Saccharomyces kudriavzevii* and *Saccharomyces cerevisiae* Grown at Low Temperature. Int. J. Food Microbiol..

[15] Belviso S., Bardi L., Bartolini A.B., Marzona M. (2004). Lipid Nutrition of *Saccharomyces cerevisiae* in Winemaking. Can. J. Microbiol..

[16] Deroite A., Legras J.-L., Rigou P., Ortiz-Julien A., Dequin S. (2018). Lipids Modulate Acetic Acid and Thiol Final Concentrations in Wine during Fermentation by *Saccharomyces cerevisiae* × *Saccharomyces kudriavzevii* Hybrids. AMB Express.

[17] Ochando T., Mouret J.-R., Humbert-Goffard A., Sablayrolles J.-M., Farines V. (2017). Impact of Initial Lipid Content and Oxygen Supply on Alcoholic Fermentation in Champagne-like Musts. Food Res. Int..

[18] Pinu F.R., Beale D.J., Paten A.M., Kouremenos K., Swarup S., Schirra H.J., Wishart D. (2019). Systems Biology and Multi-Omics Integration: Viewpoints from the Metabolomics Research Community. Metabolites.

[19] Rampler E., Coman C., Hermann G., Sickmann A., Ahrends R., Koellensperger G. (2017). LILY-Lipidome Isotope Labeling of Yeast: In Vivo Synthesis of ^13^ C Labeled Reference Lipids for Quantification by Mass Spectrometry. Analyst.

[20] Klose C., Surma M.A., Gerl M.J., Meyenhofer F., Shevchenko A., Simons K. (2012). Flexibility of a Eukaryotic Lipidome—Insights from Yeast Lipidomics. PLoS ONE.

[21] Klose C., Tarasov K. (2016). Profiling of Yeast Lipids by Shotgun Lipidomics. Methods Mol. Biol..

[22] Ejsing C.S., Sampaio J.L., Surendranath V., Duchoslav E., Ekroos K., Klemm R.W., Simons K., Shevchenko A. (2009). Global Analysis of the Yeast Lipidome by Quantitative Shotgun Mass Spectrometry. Proc. Natl. Acad. Sci. USA.

[23] Gadara D., Berka V., Spacil Z. (2024). High-Throughput Microbore LC-MS Lipidomics to Investigate APOE Phenotypes. Anal. Chem..

[24] Tarasov K., Stefanko A., Casanovas A., Surma M.A., Berzina Z., Hannibal-Bach H.K., Ekroos K., Ejsing C.S. (2014). High-Content Screening of Yeast Mutant Libraries by Shotgun Lipidomics. Mol. BioSyst..

[25] Lindberg L., Santos A.X.S., Riezman H., Olsson L., Bettiga M. (2013). Lipidomic Profiling of Saccharomyces Cerevisiae and Zygosaccharomyces Bailii Reveals Critical Changes in Lipid Composition in Response to Acetic Acid Stress. PLoS ONE.

[26] Winans M.J., Gallagher J.E.G. (2020). Metallomic and Lipidomic Analysis of *S. cerevisiae* Response to Cellulosic Copper Nanoparticles Uncovers Drivers of Toxicity. Metallomics.

[27] van ’t Klooster J.S., Cheng T.-Y., Sikkema H.R., Jeucken A., Moody B., Poolman B. (2020). Periprotein Lipidomes of Saccharomyces Cerevisiae Provide a Flexible Environment for Conformational Changes of Membrane Proteins. eLife.

[28] Hein E.-M., Hayen H. (2012). Comparative Lipidomic Profiling of *S. cerevisiae* and Four Other Hemiascomycetous Yeasts. Metabolites.

[29] Rossouw D., Bauer F.F. (2009). Comparing the Transcriptomes of Wine Yeast Strains: Toward Understanding the Interaction between Environment and Transcriptome during Fermentation. Appl. Microbiol. Biotechnol..

[30] B. Gowda S.G., Gowda D., Ohno M., Liang C., Chiba H., Hui S.-P. (2021). Detection and Structural Characterization of SFAHFA Homologous Series in Mouse Colon Contents by LTQ-Orbitrap-MS and Their Implication in Influenza Virus Infection. J. Am. Soc. Mass Spectrom..

[31] Jayaprakash J., Gowda S.G.B., Gowda D., Ikeda A., Bamai Y.A., Ketema R.M., Kishi R., Chen Y., Chiba H., Hui S.-P. (2025). Plasma Lipidomics of Preadolescent Children: A Hokkaido Study. J. Lipids.

[32] Sundaraswamy P.M., Minami Y., Jayaprakash J., Siddabasave S.G., Takatsu H., Gowda D., Shin H.W., Hui S.P. (2024). A Facile Method for Monitoring Sphingomyelin Synthase Activity in HeLa Cells Using Liquid Chromatography/Mass Spectrometry. Analyst.

[33] Jayaprakash J., B. Gowda S.G., K. Shukla P., Gowda D., Nath L.R., Chiba H., Rao R., Hui S.-P. (2024). Sex-Specific Effect of Ethanol on Colon Content Lipidome in a Mice Model Using Nontargeted LC/MS. ACS Omega.

[34] Malek M.A., Siddabasave S.G., M. Gangadhara R., Gowda D., Hui S.P. (2024). Exploration of New Lipid Nutrients and Their Characterization in Herbal Teas Using Non-Targeted Liquid Chromatography–Mass Spectrometry. Foods.

[35] Malek M.A., B. Gowda S.G., Gowda D., Hui S.-P. (2024). Analysis of Lipid Composition and Characterization of Acyl Steryl Glycosides in Rose Petals by Using Nontargeted LC/MS. J. Food Meas. Charact..

[36] Doolam B., Mishra B., Surabhi D., Mandal S.K., Sada S., Reddy N.R., Panda J., Rustagi S., Mishra A.K., Mohanta Y.K. (2024). A Systematic Review of Potential Bioactive Compounds from Saccharomyces Cerevisiae: Exploring Their Applications in Health Promotion and Food Development. Environ. Dev. Sustain..

[37] Klug L., Daum G. (2014). Yeast Lipid Metabolism at a Glance. FEMS Yeast Res..

[38] De Carvalho C., Caramujo M. (2018). The Various Roles of Fatty Acids. Molecules.

[39] Zhang H., Xu M., Shi X., Liu Y., Li Z., Jagodinsky J.C., Ma M., Welham N.V., Morris Z.S., Li L. (2021). Quantification and Molecular Imaging of Fatty Acid Isomers from Complex Biological Samples by Mass Spectrometry. Chem. Sci..

[40] Jalc D., Certik M., Kundrikova K., Namestkova P. (2007). Effect of Unsaturated C_18_ Fatty Acids (Oleic, Linoleic and α-Linolenic Acids) on Ruminal Fermentation and Production of Fatty Acids Isomers in Artificial Rumen. Vet. Med..

[41] Pinu F.R., Villas-Boas S.G., Martin D. (2019). Pre-Fermentative Supplementation of Fatty Acids Alters the Metabolic Activity of Wine Yeasts. Food Res. Int..

[42] Liu P., Ivanova-Petropulos V., Duan C., Yan G. (2021). Effect of Unsaturated Fatty Acids on Intra-Metabolites and Aroma Compounds of *Saccharomyces cerevisiae* in Wine Fermentation. Foods.

[43] Henry S.A., Kohlwein S.D., Carman G.M. (2012). Metabolism and Regulation of Glycerolipids in the Yeast *Saccharomyces cerevisiae*. Genetics.

[44] Arhar S., Gogg-Fassolter G., Ogrizović M., Pačnik K., Schwaiger K., Žganjar M., Petrovič U., Natter K. (2021). Engineering of *Saccharomyces cerevisiae* for the Accumulation of High Amounts of Triacylglycerol. Microb. Cell Fact..

[45] Sorger D., Daum G. (2003). Triacylglycerol Biosynthesis in Yeast. Appl. Microbiol. Biotechnol..

[46] Girardi Piva G., Casalta E., Legras J.-L., Tesnière C., Sablayrolles J.-M., Ferreira D., Ortiz-Julien A., Galeote V., Mouret J.-R. (2022). Characterization and Role of Sterols in *Saccharomyces cerevisiae* during White Wine Alcoholic Fermentation. Fermentation.

[47] Dong S.J., Yi C.F., Li H. (2015). Changes of *Saccharomyces cerevisiae* Cell Membrane Components and Promotion to Ethanol Tolerance during the Bioethanol Fermentation. Int. J. Biochem. Cell Biol..

[48] Chen A., Qu T., Smith J.R., Li J., Du G., Chen J. (2024). Osmotic Tolerance in *Saccharomyces cerevisiae*: Implications for Food and Bioethanol Industries. Food Biosci..

[49] Kim H.-C., Cho E.-J., Chang H.-J., Shin J.-A., Lee J.-H. (2024). Distribution of Dietary Phospholipids in Selected Agri-Foods: Versatile Nutraceutical Ingredients. Foods.

[50] B. Gowda S.G., Minami Y., Gowda D., Chiba H., Hui S.-P. (2022). Detection and Characterization of Lipids in Eleven Species of Fish by Non-Targeted Liquid Chromatography/Mass Spectrometry. Food Chem..

[51] Riekhof W.R., Wu J., Jones J.L., Voelker D.R. (2007). Identification and Characterization of the Major Lysophosphatidylethanolamine Acyltransferase in *Saccharomyces cerevisiae*. J. Biol. Chem..

[52] Merkel O., Schmid P.C., Paltauf F., Schmid H.H.O. (2005). Presence and Potential Signaling Function of N-Acylethanolamines and Their Phospholipid Precursors in the Yeast *Saccharomyces cerevisiae*. Biochim. Biophys. Acta (BBA)—Mol. Cell Biol. Lipids.

[53] Uyama T., Ikematsu N., Inoue M., Shinohara N., Jin X.H., Tsuboi K., Tonai T., Tokumura A., Ueda N. (2012). Generation of N-Acylphosphatidylethanolamine by Members of the Phospholipase A/Acyltransferase (PLA/AT) Family. J. Biol. Chem..

[54] Renne M.F., Bao X., De Smet C.H., de Kroon A.I.P.M. (2015). Lipid Acyl Chain Remodeling in Yeast. Lipid Insights.

[55] Lorenc F., Jarošová M., Bedrníček J., Nohejl V., Míková E., Smetana P. (2025). Effect of Wine Yeast (*Saccharomyces* sp.) Strains on the Physicochemical, Sensory, and Antioxidant Properties of Plum, Apple, and Hawthorn Wines. Foods.

[56] Mbuyane L.L., Bauer F.F., Bloem A., Camarasa C., Ortiz-Julien A., Divol B. (2022). Species-Dependent Metabolic Response to Lipid Mixtures in Wine Yeasts. Front. Microbiol..

